# Prevalence of Dumping Syndrome and Its Determinants Among Post-Bariatric Surgery Adult Patients at King Fahad General Hospital, Jeddah, 2019–2020

**DOI:** 10.7759/cureus.32630

**Published:** 2022-12-17

**Authors:** Ibrahim Alsulami, Ahmad Fathaldin, Thamer Alghamdi, Faisal Saud, Sultana Binyamin, Yasir Alghamdi, Rajaa Al-Raddadi

**Affiliations:** 1 Family Medicine, Al-Kamel General Hospital, Al-Kamel, SAU; 2 Family Medicine, Administration of Public Health, Jeddah, SAU; 3 Family Medicine, Ministry of Defence, Jeddah, SAU; 4 Family Medicine, Directorate of Health Affairs in Medina, Medina, SAU; 5 Community Medicine, College of Medicine, King Abdulaziz University, Jeddah, SAU

**Keywords:** bariatric surgery, dumping syndrome, prevalence, risk factors, saudi arabia

## Abstract

Background

Bariatric surgery is one of the most effective interventions for morbid obesity. Despite its benefits, unwanted consequences such as dumping syndrome (DS) have been reported following the procedure. This study aims to estimate the prevalence of DS and identify the factors associated with it in Saudi Arabia.

Methodology

This cross-sectional study collected data from patients who underwent bariatric surgery at King Fahad General Hospital, Jeddah, Saudi Arabia, in 2019-2020. A validated questionnaire was used to collect the data. The questionnaire included demographic items such as age and sex and items relating to DS such as nausea, vomiting, and palpitations. A modified version of the Sigstad diagnostic scoring system was used to confirm the diagnosis.

Results

Of the 240 investigated cases, two-thirds (67.5%) were females. The most reported symptoms were nausea (37.9%), vomiting (36.7%), desire to lie down (25.5%), restlessness (25.5%), and abdominal fullness (23.7%). Based on the modified Sigstad diagnostic scoring system, 75 (31.4%) patients met the criteria for DS. The bivariate analysis showed the prevalence was significantly higher in females (36.4%), those with university qualifications (40.3%), and those with high monthly income (62.5%) (p<0.05). Also, dietary behaviors in terms of frequency, size of meals, and drinking liquids with meals were significantly associated with the prevalence of DS, where the lowest prevalence was recorded among those consuming one small meal (10.9%), while the highest prevalence was found in those who ate more than one large meal (81.8%); also, the prevalence was significantly higher in patients who drank liquids with meals (40.8%) than those who drank liquids between meals (26.8%) (p<0.05).

Conclusion

In this single institutional study, we report a 31.4% prevalence of DS among our cohort. The predictors of the syndrome include gender, education level, monthly income, eating more than one large meal per day, and drinking liquids with meals. In the future, these predictors will be explained to patients before and after bariatric surgery to reduce the prevalence of such inconvenient syndromes.

## Introduction

Obesity has been recognized by the World Health Organization (WHO) as one of the most significant health problems worldwide; its prevalence has been increasing over the past decades despite extensive preventative efforts [1]. Therefore, bariatric surgery has become a treatment option for long-term weight loss among patients with morbid obesity and those unable to adopt dieting [2]. The bariatric clinic in King Fahad General Hospital (KFGH), Jeddah, Saudi Arabia, follows the American Society for Metabolism and Bariatric Surgery, which recommends surgery for patients who have BMI ≥40 and BMI ≥35 with at least one obesity-related co-morbidities (such as type II diabetes, hypertension, and obstructive sleep apnea), or patients can't achieve a healthy weight loss sustained for a period of time with prior weight loss efforts [3]. The main aim of bariatric surgery and especially gastric sleeve surgery, also known as metabolic surgery, is to reduce gastric capacity and the absorption surface area of the gastrointestinal tract. Bariatric surgery also indirectly affects hunger and satiety, changes food preferences, and improves energy expenditure [4,5].

A recent systematic review provided strong evidence of the safety and efficiency of bariatric surgery as a treatment option for morbid obesity, citing perioperative mortality rates not exceeding 0.2% [6]. However, the same studies recommended long-term research to investigate the postoperative consequences of bariatric surgery [6]. Dumping syndrome (DS) has been reported as a common consequence of bariatric surgery; a reviewed study pointed to prevalence rates ranging from 19% to 32% due to variations in the type of surgery, definition, and diagnostic test used [7]. Another study by Ahmad et al. concluded a prevalence of DS to be 26.5% in laparoscopic sleeve gastrectomy [8].

Due to the differences in pathophysiology, DS is classified into early and late DS. Early DS occurs within 30 minutes after eating a meal due to the quick evacuation of a dense hyperosmolar mass of food into the intestine, which causes rapid fluid changes in the intestinal lumen and the release of gastrointestinal hormones such as vasoactive intestinal peptide and serotonin hormones and, consequently, gastrointestinal and vasomotor symptoms [9]. Late DS usually occurs after one to three hours of consuming carbohydrates, referred to as incretin-driven hyperinsulinemia, resulting in hypoglycemia [10]. DS is currently clinically diagnosed using the modified Sigstad scoring system; a threshold value of >3.26 suggests DS. The diagnosis can be supported by a monitored glucose challenge, upper gastrointestinal series, or a gastric emptying study [8,9]. DS symptoms can be relieved by strict dietary adjustments, dividing the allowed size and types of food into small portions to be consumed over several meals, and not drinking fluids until 30 minutes after a meal [9,11].

A 2020 nationwide cross-sectional survey in Saudi Arabia reported a weighted prevalence rate of obesity with a BMI>30 of 24.7%, representing a significant and prevalent health problem [12]. Therefore, the main aim of this study is to estimate the prevalence of DS and identify the associated factors with DS among adult post-bariatric surgery patients at KFGH in Jeddah, Saudi Arabia, in 2019-2020.

## Materials and methods

This cross-sectional study was conducted at KFGH in Jeddah, Saudi Arabia. KFGH is a government-run hospital that provides free bariatric surgery for eligible patients. Adult post-bariatric surgery patients who underwent surgery in the period from January 1, 2019, to December 31, 2020, were considered eligible for inclusion in the study. Ethical approval was obtained from the Research and Studies Department, Directorate of Health Affairs, Jeddah, Saudi Arabia (Approval number: A01104).

The sample size was calculated using the Raosoft.com website (Raosoft, Inc., Seattle, Washington, United States). The total population size was 502 patients, the level of confidence was 95%, the margin of error was 5%, the response distribution was left at 50%, and the research team added 10% more to compensate for non-responders. The total calculated sample size was 240 patients.

The patients were selected using a simple random sampling technique. The patients’ medical records were obtained from the KFGH surgery list, and the medical records were numbered and sorted by date of surgery using an Excel spreadsheet (Microsoft Corporation, Redmond, Washington, United States). Next, random numbers were generated, and patients with the assigned numbers were included. The research team conducted a telephone interview with the participants, obtaining verbal consent after explaining the study objectives.

Data were collected using a specifically constructed questionnaire. The questionnaire was constructed after a series of discussions with a panel of experts comprising a subject specialist, a researcher, and a language expert and included demographic items and items relating to DS (symptoms and associated factors). The Cronbach’s alpha of the questionnaire was 0.85, which is considered an acceptable level of reliability. The modified Sigstad diagnostic scoring system was used to determine patients with DS. This diagnostic index is based on a weighted score for each individual symptom of DS, such as nausea, vomiting, palpitations, and the desire to lie down, A threshold value of >3.26 suggested DS [8].

IBM SPSS Statistics for Windows, Version 26.0 (Released 2019; IBM Corp., Armonk, New York, United States) was used for data entry and statistical analysis. Frequency distribution was used to describe the categorical variable, and Chi-square test was used to verify significance of the differences in the prevalence of DS among subgroups. A p-value of <0.05 was considered an indication of significance.

## Results

This study included 240 individuals who underwent bariatric surgery at our institution during 2019-2020. Two-thirds of our cohort (67.5%) were females, and the majority of our cohort was Saudi Arabian (92.1%). In terms of age, slightly less than half of our cohort (45%) was aged 40 years or older. More than half (53.7%) had a university degree, 47.1% had jobs, and most had a monthly income between 5,000 SAR and 15,000 SAR (equal to 1300 USD and 4000 USD). More details about the demographics of our cohort are presented in Table 1.

**Table 1:** Characteristics of the study group (n=240).

Characteristics	Number	Percentage
Gender		
Male	78	32.5
Female	162	67.5
Nationality		
Saudi	221	92.1
Non-Saudi	19	7.9
Age categories		
<30 years	54	22.5
30-<40 years	78	32.5
≥40 years	108	45.0
Education level		
Lower education level	35	14.6
Higher education level	76	31.7
University	129	53.7
Working status		
Not working	127	52.9
Working	113	47.1
Office work	75	66.5
Field work	38	33.6
Monthly income		
<5000 SAR	46	19.2
5000-15000 SAR	98	40.8
>15000 SAR	8	3.3
Missing	88	36.7

Gastric sleeve (86.6%) was the most common type of bariatric surgery among our cohort, and the most frequently occurring symptoms suggesting DS were nausea (37.9%), vomiting (36.7%), the desire to lie down (25.5%), restlessness (25.5%), abdominal fullness (23.7%), and dizziness (22.9%). More details about the clinical characteristics of the bariatric surgery type and the DS symptoms are presented in Table 2.

**Table 2:** Clinical characteristics of the study group (n=240).

Characteristics	Number	Percentage
Type of bariatric surgery		
Gastric sleeve	208	86.6
Mini gastric bypass	16	6.7
Gastric bypass	11	4.6
Gastric band	5	2.1
Symptoms after meals suggesting dumping syndrome		
Desire to lie down	61	25.5
Early	57	23.8
Late	4	1.7
Weakness	60	25.0
Early	55	22.9
Late	5	2.1
Sleepiness	53	22.1
Early	48	20.0
Late	5	2.1
Palpitation	43	17.9
Early	37	15.4
Late	6	2.5
Restlessness	61	25.5
Early	57	23.8
Late	4	1.7
Dizziness	55	22.9
Early	48	20.0
Late	7	2.9
Feeling warmth, sweating, pallor	37	15.5
Early	34	14.2
Late	3	1.3
Nausea	91	37.9
Early	86	35.8
Late	5	2.1
Vomiting	88	36.7
Early	83	34.6
Late	5	2.1
Abdominal fullness	57	23.7
Early	53	22.1
Late	4	1.6

**Figure 1:**
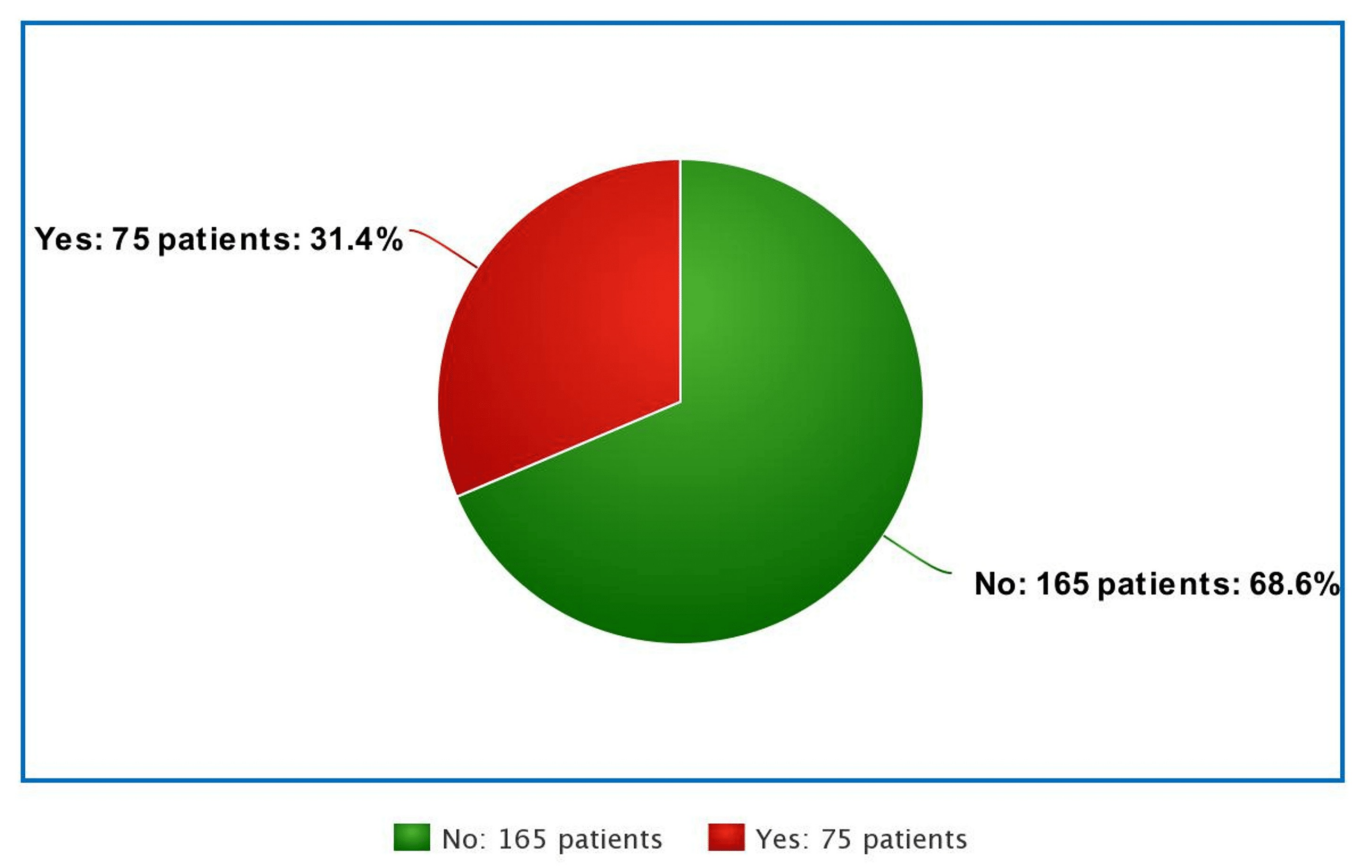
Prevalence of dumping syndrome among the bariatric surgery patients.

Based on the modified Sigstad diagnostic scoring system for DS, 75 participants (31.4%) met the criteria (threshold value of >3.26) to be categorized as DS patients. More details are depicted in Figure 1.

Bivariate analysis was performed to detect the predictors of DS in relation to the demographic characteristics of the included patients. Demographic characteristics were statistically significant among females (36.4%), those with university qualifications (40.3%), and those with monthly income >15,000 SR (62.5%) (p<0.05). More details about the bivariate analysis are presented in Table 3.

**Table 3:** Prevalence of dumping syndrome according to demographic characteristics of the patients. *Based on chi-square test; **Statistically significant

	Dumping syndrome					
Characteristics	Yes (n=75)		No (n=165)		X^2^	P*
	Number	Percentage	Number	Percentage		
Gender					6.201	0.013**
Male	16	20.5%	62	79.5%		
Female	59	36.4%	103	63.6%		
Nationality					0.300	0.584
Saudi	68	30.8%	153	69.2%		
Non-Saudi	7	36.8%	12	63.2%		
Age categories					3.049	0.218
<30 years	21	38.9%	33	61.1%		
30-<40 years	26	33.3%	52	66.7%		
≥40 years	28	25.9%	80	74.1%		
Education level					10.961	0.004**
Lower education level	6	17.1%	29	82.9%		
Higher education level	17	22.4%	59	77.6%		
University	52	40.3%	77	59.7%		
Working status					0.008	0.931
Working	35	31.0%	78	69.0%		
Not working	40	31.5%	87	68.5%		
Monthly income					12.284	0.002**
<5000 SAR	19	41.3%	27	58.7%		
5000-15000 SAR	19	19.4%	79	80.6%		
>15000 SAR	5	62.5%	3	37.5%		

Dietary behavior in terms of frequency and size of meals and the drinking of liquids with meals was significantly associated with the diagnosis of DS. The analysis showed that the lowest prevalence was among those consuming one small meal per day (10.9%), while the highest prevalence was found in those who ate more than one large meal per day (81.8%), followed by those who ate more than one small and one large meal per day (51.9%). The prevalence was significantly higher in patients who drank liquids with meals (40.8%) compared to those who drank liquids between meals (26.8%) (p-value <0.05). More details about the relationship between dietary behavior and DS are presented in Table 4.

**Table 4:** Prevalence of dumping syndrome according to dietary habits of the patients. *Based on chi-square test; **Statistically significant

	Dumping syndrome					
Meals and drinking liquids	Yes (n=54)		No (n=186)		X^2^	P*
	Number	Percentage	Number	Percentage		
Frequency and size of meals					39.608	<0.001**
One small meal	5	10.9%	41	89.1%		
One large meal	21	36.8%	36	63.2%		
More than one small meal	13	17.6%	61	82.4%		
More than one small and one large meal	27	51.9%	25	48.1%		
More than one large meal	9	81.8%	2	18.2%		
Drinking liquids					4.711	0.030**
With meals	31	40.8%	45	59.2%		
Before/after meals	44	26.8%	120	73.2%		

## Discussion

Obesity is a prevalent progressive health problem in Saudi Arabia and bariatric surgery has been recognized to be on the rise as a preferred choice for long-term reduction of weight [12,13]. Studies indicate that DS is a typical side effect of bariatric surgery, with little information about its exact frequency and factors associated with it in different settings [13]. Therefore, the current study aimed at exploring the prevalence of DS and associated factors in patients who have undergone bariatric surgery in one of the main general hospitals in Jeddah, Saudi Arabia. The modified Sigstad diagnostic score was used to define cases with a score of more than 3.26 [8]. Accordingly, out of 240 investigated cases, 75 patients (31.4%) were classified as having DS, which comes close to the upper limit of the known prevalence of DS, which ranges between 19-32%. This may be related to the degree of compliance of our patients to the dietary instructions after bariatric surgery, or it could just be, as stated by Furth et al. [7], that the differences in the reported prevalence of DS in different settings are mainly attributed to variation in definition and diagnosis of DS.

The current study revealed five main factors associated with the increased prevalence of DS; three factors related to the demographic characteristics of patients and two factors related to dietary habits after bariatric surgery and the degree of compliance of the patients with the instructions and recommendations related to dietary adjustment.

This study analyzed the demographic characteristics of DS patients and found that female participants demonstrated a significantly higher prevalence of dumping syndrome than male participants (36.4% vs. 20.5%, p = 0.013). The reason for the high prevalence of this syndrome may be due to the fact that there were more female patients than male patients in the cohort. Interestingly, Banerjee et al. found a similarly high prevalence among the female cohort in their study [14]. Moreover, there was no difference between the age group and the presence of DS in the present study. This goes in line with previously published data, as Ahmad et al. too did not find any correlation between the age of the patients and the development of DS [8]; however, Banerjee et al. suggested that further studies seeking such correlation are recommended [14]. Furthermore, as for the educational level and monthly income of the patients, the most affected group was the university-level educated and higher-income patients, respectively. There are no previously reported data correlating these two factors and the presence of DS. The authors hypothesize that this result may be due to confounding factors, as the number of enrolled patients that are in university or obtained a university degree was higher. In contrast, the higher income group was small and may not reflect the characteristics of the general population.

The fourth factor was noncompliance to the given instructions regarding the frequency and size of meals after bariatric surgery [11]; where the highest prevalence of DS was reported by patients who ate more than one large meal after bariatric surgery compared to those who divided the allowed quantity of food into small portions consumed as several meals. The explanation of this difference could be viewed under the pathophysiology of the syndrome; especially the early DS [9,15]. Typically, the food is partially digested in the stomach before moving to the duodenum under the control of the pyloric sphincter, which allows only for the gradual passage of small particles; the pyloric tone as well as the feedback from the duodenum control the rate of gastric emptying [15]. In bariatric surgery, due to altered gastric anatomy and innervation of the pylorus, uncontrolled large hyperosmolar food particles move rapidly to the intestine, leading to the shifting of fluids from the intestinal vasculature to the intestinal lumen; which are responsible for the resulting gastrointestinal symptoms and vasomotor symptoms identified in previous studies [10,16], as well as the current study. From these sequences of events according to the pathophysiological explanation, it is easy to understand why the large meals resulted in a significant increase in the likelihood of encountering DS.

The fifth factor associated with DS identified in the current study was drinking liquids with meals; the prevalence of DS was found significantly higher in patients who drank liquids with meals compared to those who drank between meals. Again, this notion could be explained in light of the pathophysiology of DS, where Ukleja stated that “The accelerated gastric emptying of liquids is a characteristic feature and a critical step in the pathophysiology of DS” [15], and drinking liquids with solid food enhances rapid emptying of the stomach into the intestine and exaggerate dumping [16].

The main limitation of the current study is that the data is based on subjective responses; for example, the definition of the size of the meal (small or large), which are broad terms subjected to remarkable variations between individuals.

## Conclusions

This study aimed to measure the prevalence of DS and to report the predictors of this complication following bariatric surgery. More than 20% of the 240 patients who underwent bariatric surgery in the study period suffered from DS. The predictors include eating more than one large meal per day and drinking liquids with meals. These predictors must be considered and discussed thoroughly with patients to reduce the occurrence of DS.

We recommend that the management of bariatric surgery cases starts before the surgery with dietary and behavioral education, and stress the necessity of close collaboration after the surgery of multidisciplinary specialties such as gastroenterologists, surgeons, family physicians, and nutritionists.

## Figures and Tables

**Figure 2:**
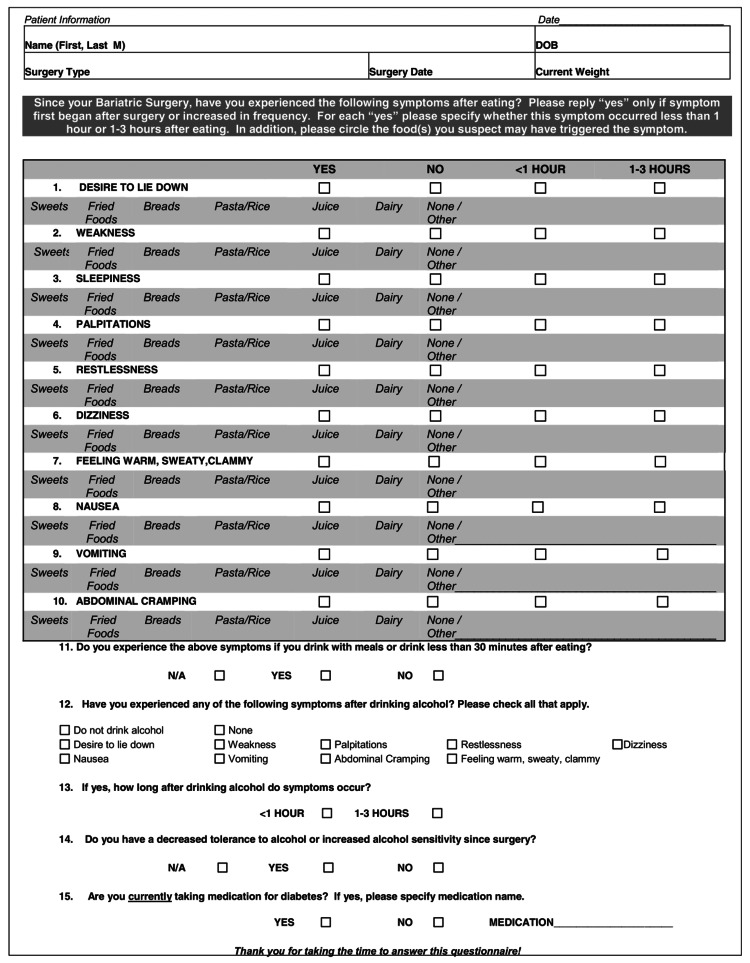
The Modified Sigstad Scoring System
