# Correction: Prevalence of Dumping Syndrome and Its Determinants Among Post-Bariatric Surgery Adult Patients at King Fahad General Hospital, Jeddah, 2019–2020

**DOI:** 10.7759/cureus.c466

**Published:** 2026-07-24

**Authors:** Ibrahim Alsulami, Ahmad Fathaldin, Thamer Alghamdi, Faisal Saud, Sultana Binyamin, Yasir Alghamdi, Rajaa Al-Raddadi

**Affiliations:** 1 Family Medicine, Al-Kamel General Hospital, Al-Kamel, SAU; 2 Family Medicine, Administration of Public Health, Jeddah, SAU; 3 Family Medicine, Ministry of Defence, Jeddah, SAU; 4 Family Medicine, Directorate of Health Affairs in Medina, Medina, SAU; 5 Community Medicine, College of Medicine, King Abdulaziz University, Jeddah, SAU

This article has been corrected to fix multiple discrepancies in reported totals and percentages throughout the article, and update the IRB approval number. These changes do not alter the underlying dataset, statistical analyses, or the direction, magnitude, or interpretation of the study findings.

